# Emotion-Focused Therapy for Autism Spectrum Disorder: A Case Conceptualization Model for Trauma-Related Experiences

**DOI:** 10.1007/s10879-018-9383-1

**Published:** 2018-02-17

**Authors:** Anna Robinson

**Affiliations:** Centre for Autism, School of Education, University of Strathclyde, 636b, Curran Building, 141 St James Road, Glasgow, G4 0LT Scotland, UK

**Keywords:** Emotion-focused therapy, Autism spectrum disorder, Group therapy, Trauma-related experiences, Case conceptualization

## Abstract

People with autism spectrum disorder (ASD) report painful experiences through emotional misunderstandings with typically developing peers. There are limited intervention methodologies for ASD on the impact of emotional injuries and how to work with resulting trauma. This paper presents a rational-empirical model of trauma-related experiences with the first presentation of a new case conceptualization model for emotion-focused therapy for ASD. It describes the transformation of problematic emotion schemes through a sequence of emotional processing steps illustrated with a case example. These steps include: overcoming differentiation of core painful feelings (such as loneliness, shame, and fear); autobiographical memory recall of distanced trauma, using a novel method of video Interpersonal Process Recall; and articulation of the unmet needs contained in core painful feelings. This is followed by the expression of an emotional response to those feelings/needs; typically, self-soothing, protective anger and compassion responses offered interpersonally by group members. These emerging adaptive emotions facilitate mentalization of self and other that strengthens intrapersonal and interpersonal agency. This rational-empirical case conceptualization acts as a hypothesis for testing in subsequent trials.

## Introduction

Autism spectrum disorder (ASD) is a neurodevelopmental disorder characterised by social communication difficulties and restricted repetitive thinking and behaviour (American Psychiatric Association 2013). ASD is also associated with difficulties regulating emotion and coping with stress (Mazefsky et al. 2013). Further, up to 85% of individuals with ASD present with alexithymia (Hill and Berthoz 2010) as well as a general tendency to intellectualize rather than experience and process emotions (Mazefsky and White 2014). Individuals with ASD are generally believed to lack empathy (Gillberg 1992). However, research indicates that individuals with ASD possess difficulties in self-understanding or theory of own mind (Williams 2010) as well as theory of mind (Baron-Cohen 1997) but not with affective empathy (Dziobek et al. 2007). Consequently, people with ASD experience higher levels of emotional distress and additional mental health difficulties compared to typically developing (TD) peers. Research indicates that 74% of young people with ASD had clinically significant emotional difficulties, such as anger, sadness or anxiety, compared to 18% of TD peers (Totsika et al. 2011).

Cognitive and behavioural therapies are the most studied treatments for individuals with ASD and the preferred psychological treatment for coexisting anxiety disorders (National Institute for Health and Care Excellence; NICE, 2012). Recent systematic and meta-analysis have shown that modified CBT produce small to moderate effect sizes when based on informant or clinician outcome measures (Spain et al. 2015; Weston et al. 2016), with a small and non-significant effect size when using self-report measures. Although CBT produce encouraging results, and continues to evolve, some researchers have emphasized that there are still many individuals with ASD who do not respond well to CBT or who remain considerably symptomatic at the end of treatment (Weston et al. 2016).

Adaptations of CBT interventions for adults with ASD are recommended and these include a more concrete, structured, visual approach that targets changing behaviours rather than cognitions. The recommended NICE guidance for interventions for core symptoms of ASD is a psychosocial group-based learning programme focused on improving social interaction. These should typically include methods such as modelling, with explicit rules that teach suggested strategies for dealing with socially difficult situations and should incorporate peer feedback with discussion and decision-making. These approaches do not address core processes such as emotional cognition and empathy. An emerging alternative that does address these core processes is humanistic-experiential psychotherapy (HEP) with a diverse evidence base (Elliott et al. 2013).

One such HEP is emotion-focused therapy (EFT) that has a growing evidence base for conditions such as depression (Greenberg and Watson 2006) social anxiety (Elliott 2013; Shahar 2014) and generalized anxiety disorder (Timulak and McElvaney 2016) as well as complex trauma (Paivio and Pascual-Leone 2010). Emotion-focused therapy for complex trauma (EFTT; Paivio and Pascual-Leone 2010) is a short-term treatment for childhood abuse and neglect that proposes the therapeutic relationship and emotional processing of trauma material as key mechanisms of change. These trauma feelings and memories are accessed so they are available for exploration and change (Foa et al. 2006). The main focus of EFTT is the resolution of past issues utilizing an empty chair task to facilitate working through unfinished business with particular perpetrators of abuse and neglect, usually attachment figures. The focus of current methodologies is primarily on teaching adaptive skills whether this be addressing core ASD differences or reducing the impact of comorbid mental health symptoms. There are however, limited research or intervention methodologies for ASD on the impact of emotional injuries and on how to work with resulting trauma.

Trauma includes a real or perceived threat of physical harm or sexual violence (American Psychiatric Association 2013). Children with disabilities are considered a vulnerable population (Cohen and Warren 1990) and are at greater risk of victimization than those without disabilities (Rand and Harrell 2009; Sullivan 2009). Research indicates they are 4–10 times more likely to be victims of violence, abuse, and/or neglect (Petersilia 2001). The prevalence of trauma and related symptomology in ASD is unknown. There is however, growing recognition that individuals with ASD are at increased risk to experiencing and be detrimentally affected by traumatic childhood events (Kerns et al. 2015). Emotion recognition and insight play a key role in emotion regulation ability and the processing of trauma (Jones et al. 2011). Some people with ASD report being disconnected from their emotional experiences (Gerland 2003). This inability to access one’s emotional life can prevent recognizing and effectively coping with traumatic experiences.

**Fig. 1:**
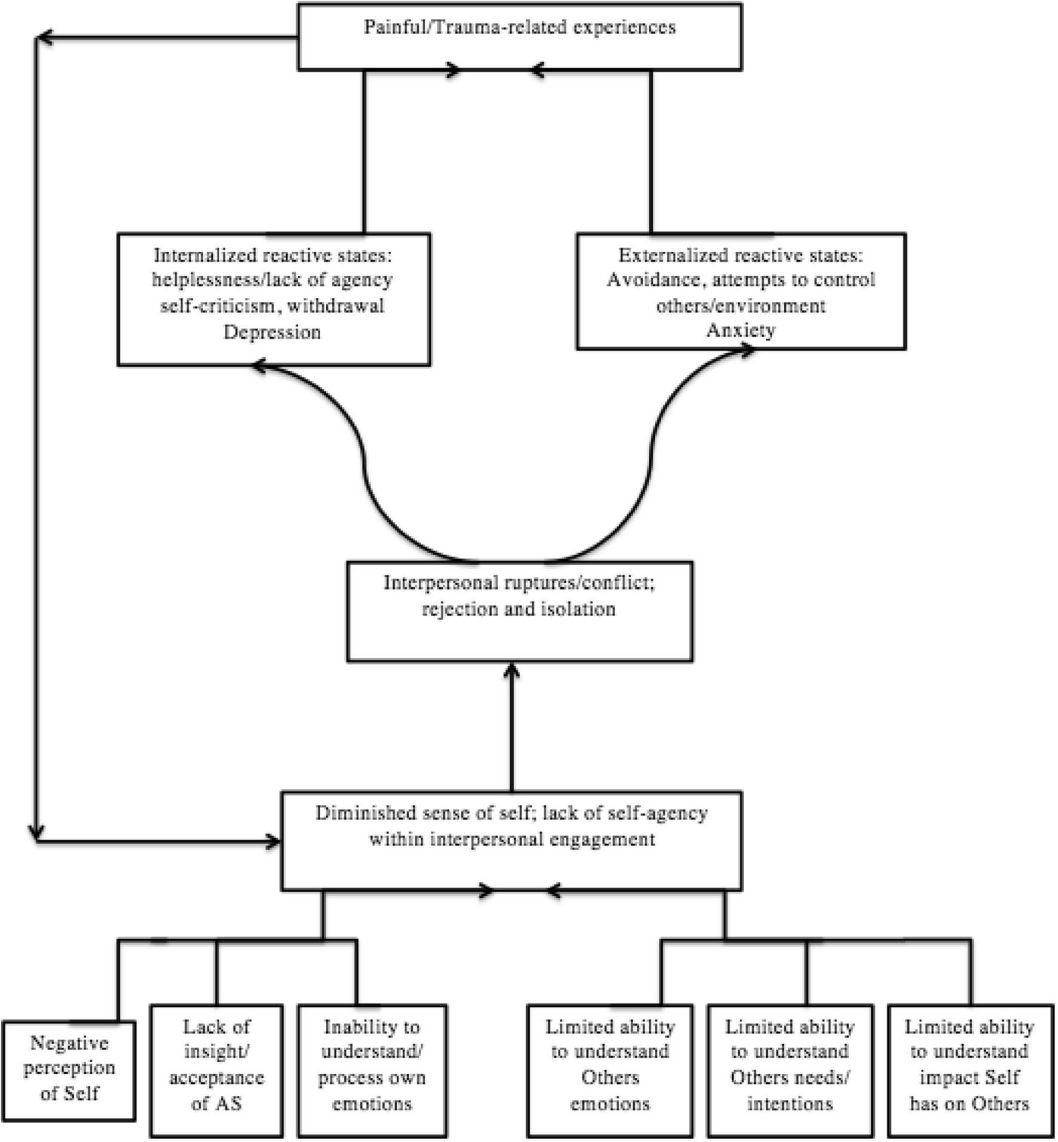
Rational-empirical model for diminished sense of self and interpersonal relatedness difficulties leading to trauma related experiences

Having a mechanism to understand trauma-related experiences in ASD is a useful starting point and a rational-empirical model based on a grounded theory analysis (GTA) of 43 interviews with parents living with adolescents coping with ASD is proposed (see Fig. 1). From the GTA of these 43 parental accounts a core-unifying category emerged that social-emotional relational differences predispose adolescents with ASD to developing trauma-related experiences. The social-emotional processing differences of adolescents with ASD manifest a fragile sense of self and lack of self-agency within interpersonal engagement that lead to interpersonal ruptures, rejection by others and social isolation. This results in both internalized and externalized reactive responses that subsequently lead to trauma related experiences. It is hypothesized that youth with ASD are predisposed to developing trauma-related experiences due to repeated negative encounters with TD peers within non-accessible social environments.

Recent findings indicate individuals with ASD manifest autobiographical memory difficulties characterized by reduced specificity, less elaborated and detailed self-narratives with lower coherence, and an increased reliance on scaffolding for retrieval (McDonnell et al. 2017). These autobiographical memory difficulties consist of episodic elements including memory for personally experienced events contextualized within one’s sense of self over time (Conway et al. 2004). Therefore, supporting recall and reprocessing of autobiographical memory and strengthening this fragile sense of self and one’s self-agency within interpersonal relationships are central for therapeutic change (Robinson and Elliott 2017).

Preliminary positive findings for Emotion-Focused Therapy for Autism Spectrum (EFT-AS; Robinson and Elliott 2017) have been reported. Due to the heterogeneous profile of ASD we can expect a varied response to trauma, making it difficult for therapists to identify signs of trauma in clients with ASD. Furthermore, there is limited research on how people with ASD process traumatic events and incorporate these within their autobiographical memories. Specifically, there are limited accounts of how clients with ASD work with trauma-related experiences in therapy. In view of this absence, this paper proposes a preliminary model of how group EFT-AS can deepen experiential processing enabling access to trauma-related experiences so they can be available to transformation in therapy. First, a brief overview of group EFT framework for ASD is provided (for a description see Robinson and Elliott 2017). This is followed by the first presentation of a case conceptualization model of working with trauma-related experiences in therapy for ASD. This case conceptualization has been developed by the author in a currently running program of research into the development of EFT for ASD and its potential to shed light into how we might work with trauma.

### EFT Framework for Autism Spectrum Disorder (EFT-AS)

EFT-AS is a small group version of EFT. Similar to individual EFT, it is marker driven with the therapist identifying markers that indicate a client’s readiness for certain therapeutic tasks. EFT-AS adopts a phenomenological approach organized around autistic process (AP) markers. These AP markers are selected from the therapy session using video assisted interpersonal process recall (IPR) and played in the subsequent IPR session. This cycle of therapy followed by IPR session repeats in cycles until the final ending session. In EFT-AS, IPR is used as a clinical process-guiding method to facilitate client awareness and arousal. Specifically, to scaffold areas of difficulty related to affective empathy for self (emotional self-attunement or processing own emotions) and for other (interpersonal attunement or empathic relating) and also areas of difficulty related to cognitive empathy for self (self-understanding, reflection and conception or theory of own mind) and for other (theory of your mind or mental representation). Further, video IPR can scaffold retrieval of autobiographical memory recall, can act as a visual concrete *part of self* (e.g. fragile self) for empty-chair work, initial step to focusing to deepen client experiencing and evoke emotional responses to self to support emotion regulation difficulties.

The following presents of an emerging case conceptualization model for working with trauma-related experiences within an EFT framework for clients with ASD. To reflect a more HEP stance, the terminology of this case example will use client with autistic process.

## Emotion-Focused Case Conceptualization Model for ASD

**Fig. 2:**
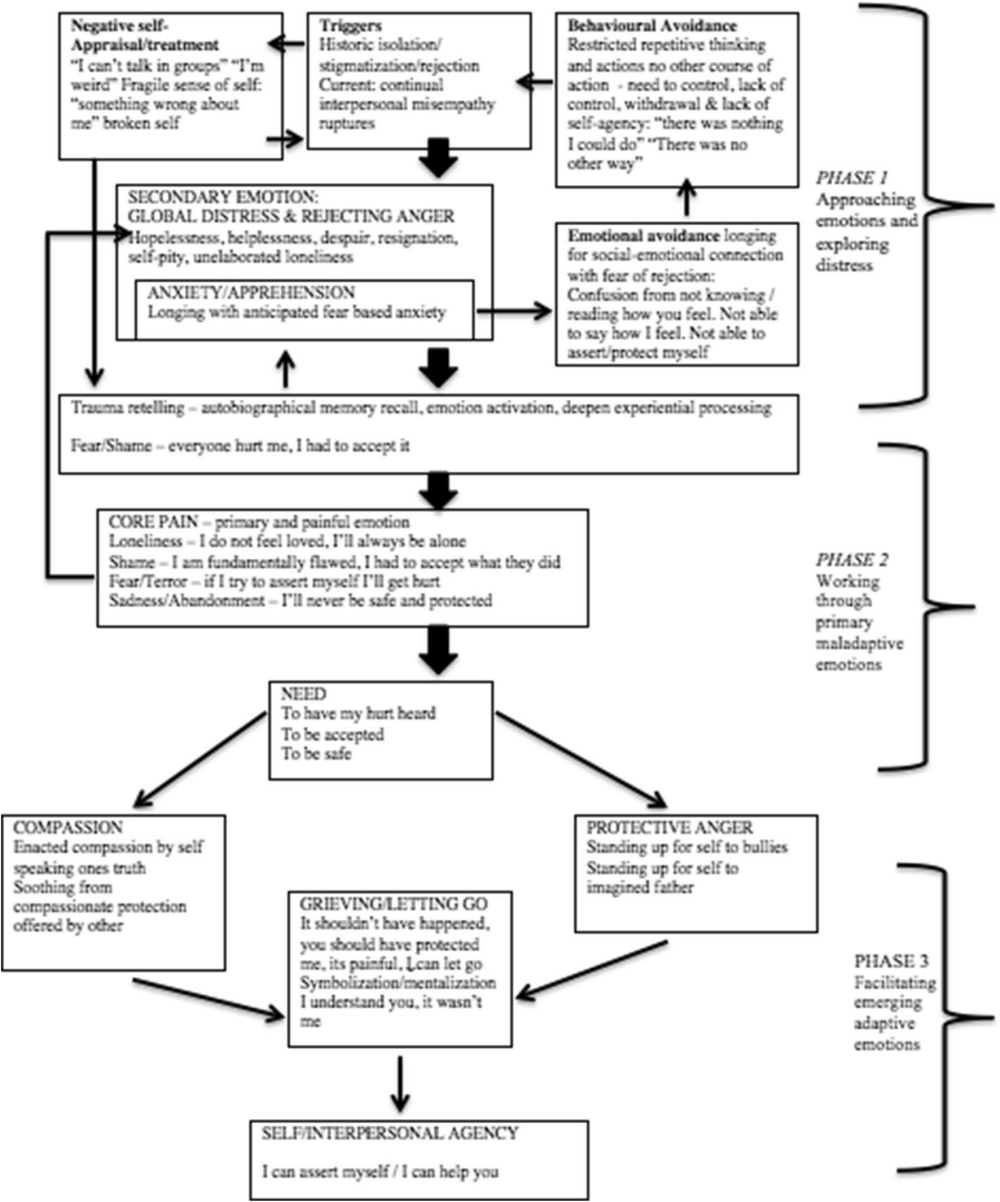
The model of Martin’s case conceptualization and emotion transformation in therapy

EFT draws upon a Task Analytical methodology to study psychotherapeutic change within a discovery and validation phase (Greenberg 2007; Pascual-Leone et al. 2009). The discovery-oriented phase of task analysis involves the construction of a specific model of client change process and a method of measuring its components. A rational model is constructed and acts as a record against which future observations can be checked (Greenberg 2007). This ASD case conceptualization (Robinson 2017) has been guided by the framework initially developed on research on emotional transformation in experiential treatment of depression (Pascual-Leone and Greenberg 2007) and specifically for Generalized Anxiety Disorder (Timulak and Pascual-Leone 2014). To the best of my knowledge this is the first presentation of a rational-empirical model of working through trauma related experiences for ASD (see Fig. 2). This rational model acts as a hypothesis for testing in subsequent trials (the author is currently leading a program of research for EFT-AS).

The case conceptualization model (see Fig. 2) presents a clinical strategy and actions of a therapist working with clients with autistic process. The strategy consists of several aspects within three phases leading to emotion transformation. The therapist uses this case conceptualization framework to inform the dynamics of the client’s distress (see phase 1 Fig. 2) using this to orient identification of triggers and self-treatment, overcoming avoidance and focusing on the underlying core painful emotions. Following this, the therapist supports the client to articulate the unmet need (see phase 2 Fig. 2). For the client with autistic process this can involve access to distanced trauma related experiences, evoking and activation of emotions associated with the event and experiential processing of these emotions.

Once the client is able to access their core pain and to articulate the associated unmet need, the next step for the therapist is to facilitate emerging adaptive emotions (see phase 3 Fig. 2), such as compassionate responses to self. In conjunction with an empathic relational stance, as EFT-AS is a group therapy, the therapist has the potential to facilitate compassionate responses from other group members. In addition to compassion, assertive and protective anger is generated in well proceeding EFT cases (Timulak and Pascual-Leone 2014). In EFT, it is seen as an important step in the transformation of emotions that the client feels the anger in the moment.

The therapist seeks to facilitate both self-compassion and assertive, protective anger. This is achieved through enactment tasks, such as an imaginary dialogue of a situation in which the client can speak their truth to a significant other (e.g. a lost parent) or express their anger to a harmful other (e.g. abusive bully). The therapist can use IPR to scaffold barriers to the imagination required when working with multiplicity or parts of self through two-chair enactment tasks. Specifically, in EFT-AS the visual image of self can be on screen and the client can speak to their fragile or broken younger self from the position of stronger adult self. Research indicates that once self-compassion and protective anger have been achieved and expressed, a grieving process can begin (Pascual-Leone and Greenberg 2007; McNally et al. 2014) as shown in the final phase of the case conceptualization model (see phase 3 Fig. 2). Finally, these emerging adaptive emotions facilitate mentalization of self (theory of own mind) and other (theory of your mind) that strengthens intrapersonal and interpersonal agency. This case conceptualization is illustrated in the following case example.

### The Case of Martin

Different parts of this case conceptualization interplay with each other and are co-constructed with the client. As EFT-AS is a group format there is an evolving group dynamic that is also co-constructed as clients share their life narratives in-session and across treatment. This case example is taken from a small group EFT-AS carried out by the author over 11 weeks. The group consisting of three adults, two males and one female, in their late thirties and early forties, all diagnosed in adulthood with Asperger Syndrome (AS). All three adults were formally diagnosed with AS by specialist ASD clinician within diagnostic teams according to Diagnostic and Statistical Manual-IV. The focus here is on Martin one of the male participants. Martin’s scores using the Client Emotional Processing Scale for Autism Spectrum (CEPS-AS; Robinson and Elliott 2016) for emotion processing, self-reflection, empathy and mental representation changed from low level processing at the beginning to moderate to high level processing at the end of treatment.

### Identifying the Presenting Problem

The initial step in EFT case formulation is to identify the client’s presenting problem through the client’s narrative (Elliott et al. 2004; Goldman and Greenberg 2015). Clients with autistic process may locate their own difficulties in global ASD terms (e.g. as difficulties talking to people) whilst others may find locating a therapeutic focus difficult. For example,

- Therapist: And you Martin, what would you like to focus on over the weeks?
- Martin: Erm [puts head down] Erm [long pause] …
- Carla: You’ll think of lots of things when you get outside

## Triggers of Emotional Pain: Misempathy Ruptures

The therapist is listening for client triggers that are current, interpersonal situations that trigger underlying painful emotions, such as isolation or rejection. A problematic relational problem that people with autistic process report is having their emotions misunderstood by others. Therapists are looking for emotion markers to identify and change negative interaction cycles. This can be described as a form of misempathy (emotional misunderstanding). These markers can occur as interpersonal ruptures in the session between group members or through client dialogue of past negative interpersonal experiences. Misempathy is a trigger within EFT-AS and is often reported by most clients. Martin referred to a number of historical as well as current triggers that brought him distress. Specifically, the therapist identifies emotion misunderstanding dialogue that indicates a client believes their emotional intentions have been misunderstood by others. For example,

- Martin: I got pulled upstairs by a supervisor I worked with for doing something wrong, yeah, they did say I’d been confrontational and aggressive and they did use those words when I was trying to be assertive. So they didn’t understand me.

It is proposed that triggers of problematic emotional processing in ASD are current situations that trigger underlying painful emotions that resemble interpersonal ruptures that have occurred throughout childhood, adolescence and persist into adulthood. These are chronic recurring social-emotional patterns of failed interactions that led to developmentally significant emotional injuries (see Fig. 1). These originate from core ASD difficulties of social-emotional processing through self-understanding, such as emotion regulation, self-reflection or theory of own mind and other-understanding, such as empathic attunement and mentalization or theory of mind. The triggers are actions or perceived actions of significant others (i.e. peers) such as rejection (i.e., exclusion, invalidation and humiliation). Emotional schematic processing builds on memories of unsuccessful processing of difficult situations, leading to chronic maladaptive emotional experiences (Greenberg 2011). Observing current triggers bringing painful emotional experiences reported by clients with autistic process, in clinical practice, it appears that many of these triggers resemble the ones reported by clients with depression and social anxiety. In fact, clients with autistic process have significantly higher rates of comorbid anxiety and depression then the typical population. They include developmentally delayed social communication and interaction skills, followed by repeated and chronic, failures to socially interact and a perception of repeated and chronic, hurtful actions usually by peers, such as rejection, exclusion and stigmatization. Furthermore, these tend to be associated with failures by adults to prevent such treatment.

## Secondary Emotions—Global Distress

Potential threats (triggers) are all pervasive for a person who has a desire to relate to others but is in a state of interpersonal confusion and constant fear of rejection. Furthermore, efforts to avoid triggers or cope with them do not avoid the emotional pain but on the most part, lead to exhausting longing with persistent sense of inability and fearful apprehension. Thus, clients with autistic process present with a sense of not belonging or fitting into the world, of hopelessness, helplessness, desperation and annoyance at self for social inadequacies and fear of interpersonal encounters. These emotions are secondary to more primary unbearable and unprocessed core painful feelings. They are typified by low differentiation of various feelings and an overall global sense of being distressed.

The therapist should be attuned to key repeating triggers of client problematic process that point to the underlying determinants of their difficulties. An example of an autistic process (AP) marker is identifiable by the client’s expression relating to a lack of self-agency within interpersonal relationships. This can be seen when Martin shares his rationale for moving to University so he can develop social relationships. Martin spoke of how he left his job to go to University because he could not get a social life after leaving school. He wanted friends and thought by going to University this would increase his chances of succeeding at this. For example,

Martin: I found that, you’re supposed to have a balance of a social life and work life. I couldn’t get that balance. Well, I couldn’t get any social thing out of it really, so I ended up not studying anything. […]

Martin: When I was, when I worked straight from school it was the same, that’s probably why I left to go to University I was hoping for something to change but it didn’t happen. I was hoping for change I thought this would be a chance it just got worse because my expectations were too high.

## Negative Self-Treatment

Through observation, clients with autistic process attempted to deal with distress or potential distress by controlling their own actions (withdrawal), others behavior (dominant controlling actions) or their environment (see Fig. 1). They engage in repetitive thinking by punishing themselves, or being negative towards the self whilst preparing for the distress of rejection. In the context of problematic triggers (e.g., failed social interactions with negative judgment by others) the clients could be preparing the self (e.g., its never going to happen) by beating themselves up or preventively judging the self (e.g., I’m not capable) and in this way trying to improve their own coping or at least prepare themselves for the pain of rejection. Typically, they might make the self responsible for any potential negative interaction (e.g., I can’t talk to people, I can’t say things the right way). There can also be genuine dislike and non-acceptance of self (e.g., I’m weird, I’m not normal). In accordance with Timulak and Pascual-Leone (2014) it is hypothesized that negative self-judgment can be an introjected criticism from others. Further, it is hypothesized, that for clients with autistic process this negative self-judgment manifests as a fragile sense of self as a consequence of concrete experiences of habitual failed interpersonal encounters. These are based on their social interaction differences that occur in childhood with TD peers and significant adults that persist into adolescence and adulthood.

The case conceptualization begins at phase 1 (see Fig. 2) with approaching emotion and exploring distress as it involves deepening the clients experience and facilitating self-awareness of triggers and differentiating global distress. The therapist identifies autistic process (AP) markers, selects and edits these for video IPR and plays these in the following recall session. These IPR clips have multiple markers for multiple clients and they are offered to the group. In session, the therapist plays IPR selected moments of negative self-treatment, fragile sense of self or the self-critic and uses these to deepen the clients self-experience through an empathic relational stance and for setting up intrapersonal and interpersonal therapeutic work. For example,

[part of an IPR edited clip]

Therapist: How did that make you feel? That you had gone to University, you’d wanted friends and it didn’t seem to happen?Martin: I felt that there was something not right about me. Something wrong. I just didn’t think there was anything to do and that there was no point in trying anymore, so I became more isolated and stuck in my flat on my own.

## Anticipatory Anxiety, Emotional and Behavioral Avoidance

### Longing with Associated Rejecting Fear

Feared triggers and the emotional pain they bring are also controlled through emotional avoidance mainly through a hidden longing desire to connect, but fear based shame and corresponding behavioral avoidance. This lack of understanding of how to socially and emotionally read TD others, generate an apprehensive anxiety. This is often associated with growing up sensing being different, but no understanding of this until a late adult ASD diagnosis. This often generates a general undifferentiated sense of shame and negative self-perception. The therapist looks for these global signs of distress and uses this as an emotion compass (Greenberg and Watson 2006) to point to the underlying painful emotion or core pain. For example,

- Martin: I ended up having to leave; I ended up having to leave there, [smiles and quiet laugh] I couldn’t go on
- Therapist: Martin you smiled there

Through the on going development of a safe relationship and offered empathy Martin is able to start to explore his experiences of being bullied. Initially, these are expressed with flat emotion and a sense of self-resignation. Exploring these and viewing self, sharing these experiences through the use of IPR there is a slow unfolding of distanced trauma through chronic peer victimization. For example,

- Martin: I was horrendously upset by bullying when I was a child. Although I was still getting called the names in my adulthood.
- Martin: Even teachers, even a teacher called me the names […]
- Martin: I got bullied at the golf course, for being thin and I got called these girls names, I got bullied because I was quiet, bullied because people thought they could get away with it.

## Retrieval of Distanced, Not Recognized Trauma

Creating narratives about oneself requires internal coherence (Pascual-Leone 1990) a particular challenge for people with autistic process as they have autobiographical memory difficulties characterized by reduced specificity, less detailed self-narratives with lower coherence (McDonnell et al. 2017). Further, there is currently limited research to the extent that people with autistic process experience and emotionally process coherent narratives relating to trauma. These particular issues with trauma retelling mean that trauma memories may be distanced or may not be identified as trauma. Therefore, trauma retelling usually begins with the current trigger of avoidance of people, of not getting it right or of being misunderstood by others.

The therapist uses this presenting issue or undifferentiated global sense of helplessness and selects this for video IPR to play in the following session. This is used as a process-guiding task within trauma retelling to expand and specify autobiographical memory recall, to evoke emotion activation usually for overregulated emotion states, scaffold the imagined two-chair enactment task by showing the fragile self part visually and to deepen experiential processing.

During the session there was a slow recall of the memory as Martin processed the distanced experience with a lack of self-agency and resigned despair. For example,

- Martin: I could have used violence on all those people at the golf calling me names. I had no chance at using violence against them because I was so small and thin and I wasn’t very strong […]
- Martin: I wish I had responded and said something against them, but it probably would have gone against me

## Reaching Core Emotional Pain

In EFT, the core pain is the underlying primary painful emotional response to the triggering situations or perceived trigger (Timulak and Pascual-Leone 2014). The term core pain is used as it indicates to the therapist that these painful emotions should be the focus in therapy. At the core of the ASD dynamic, there are emotion schemes centered on painful emotions that are pervasive, overwhelming and painful that in part are primary maladaptive emotions (Greenberg 2011) as they do not serve any adaptive action and thus the client wants to avoid them. However, autism is often referred to as a hidden disability with associated limited capacity to read the emotions and intentions of others, this means they can be vulnerable to victimization from others. Therefore, in part there is some aspect of ‘logical self-protection’, but when these are coupled with restricted repetitive thinking can become entrenched thought patterns. Hence, why a psycho-education approach to teach social skills is often the preferred course of treatment for people with ASD. From observations in clinical practice, this approach does not treat the underlying core pain and emotional injuries.

The case conceptualization moves to the second phase, by working through primary maladaptive emotions (see Fig. 2). It is hypothesized that for ASD emotions are shame-based (e.g., I am broken, defective), loneliness/sadness based (e.g., I feel alone, I’ll always be alone), and fear/terror-based (e.g., I am frightened of you, you’ll reject, attack or hurt me). Once the trauma and chronic associated feelings have been accessed by the client with autistic process, the underlying core painful feelings centers on a profound sense of loneliness, shame and traumatic fear.

Varying degrees of resolution involve clients reaching their core pain and using it to better understand self and others. The primary goal of the therapy is to process the presenting maladaptive emotions that diminish the self, such as self-loathing and passive, hopeless despair. Specifically, EFT-AS promotes in-session experiencing of emotion with the goal of fostering, with the supportive guidance of the therapist, an acceptance of experienced emotion, a capacity and proficiency in regulating emotion and in self-soothing, and a transformation of destructive or ‘maladaptive’ emotions to more healthy alternatives. For example,

- IPR CLIP [Martin talking about being bullied]

In recall, the therapist deepens the experience to move towards the core pain.

- Therapist: When you see yourself Martin how do you feel
  Martin: I did feel like it was too late like I said, I do feel like it’s too late in some ways
- Therapist: Too late…
- Martin: For recovery
- Therapist: You feel as if there is no way for you to be ok, to recover
- Martin: Maybe what I mean by recovery is that I’ll never be normal. That’s one thing maybe…. [lowers voice and puts head down] I’ll never have a relationship, [shakes head] I’ll never be included …. [holds head down]
- Therapist: As if you couldn’t see yourself in a different place
- Martin: I’d be stuck; I’d be stuck, yeah. I’d still always lack confidence, I’d still always lack confidence…I’d always be alone

## Discovering the Unmet Need

In EFT, research indicates that for depression, trauma and anxiety certain types of needs are associated with certain clusters of primary and painful emotions (Timulak and Pascual-Leone 2014). These experienced core pain indicates that the client’s needs are not fully met (Greenberg 2011). The needs corresponding with shame-based emotions include, the need to be seen, included and acknowledged. The needs corresponding with loneliness/sadness-based emotions include, the need to be connected, cared for, be liked/loved. The needs corresponding with primary terror/fear-based emotions include, the need for safety and protection, control and self-agency.

This begins the process of discovering Martin’s core pain and this stems from his feelings of desire for, but never having any friend and relationship, to feelings of deep sadness through loneliness. Once we reach this core pain this is further explored to discover additional associated core pain, with the aim of facilitating emotion transformation in the final phase of therapy (see phase 3 Fig. 2).

## Transformation of Emotional Pain

One of the core principals of EFT is you have to arrive at an emotion (core emotion) and change this with an emotion (Pascual-Leone and Greenberg 2007). Therefore the key to successful task resolution is emotion transformation. In Martin’s case conceptualization he experienced trauma as a result of chronic bullying, which was evoked as a distanced vulnerability during the second phase of therapy. This led to experiencing the anger in session that moved towards assertive protective anger for self. For example,

Martin: The annoying thing was that the adults were calling me the name as well, so the anger that that generates is still there... […]

- Martin: I could viciously attack them all […] I could viciously attack them if I wanted for calling me the name […] …No, I didn’t, but I would, I feel like doing that now […]
- Therapist: There is strength in your voice. It sounds, a strong anger
- Martin: I am angry with all those people who called me the name is if that was ok, as I accepted it. I didn’t
- Therapist: If you could tell those people who called you those names what would you say?
- Martin: It wasn’t ok, that wasn’t my name. The teacher should have stopped it. My sister should have stopped […]
- Therapist: That wasn’t my name
- Martin: I feel like asking my sister why did you let him speak to me like that?

In EFT, clients engage with enactment tasks which involve dialogue with different parts of self or imagined significant others, such as a parent or an abusive other. These tasks, as well as an empathic relational stance, are the main means of accessing emotions to make them available for exploration and transformation. Thus, adequate processing of sadness at loss and anger at violation often form the adaptive core of the treatment of both depression and anxiety (Greenberg 2011). This can present a challenge for clients with autistic process who have cognitive empathy difficulties, such as self-awareness or theory of own mind (Williams 2010) and mentalization of others mind (Baron-Cohen 1997) as well as restricted repetitive thinking. However, an enactment task (unfinished business) was explained to Martin and he was able to respond to his own core pain of sadness at his feeling of abandonment by his dead father. For example,

- Therapist: In EFT we sometimes use [explanation of enactment task]…If you could speak to your Dad, if he was here in front of you, sitting in that chair, what would you like to say to him?
- Martin: What?
- Carla: You’ll never get closure that way [therapist puts hand up towards Carla to signal for her to let this task with Martin continue]
- Therapist: When we can’t speak to someone because they are no longer alive we can have an imagined conversation as if they are here in front of you. So, if your dad was in that chair in front of you. Let’s put him there [points to empty chair] what would you want to say to him?
- Martin: I’d just ask him why didn’t you stand up for me? … [lowers head]
- Therapist: [lowers tone of voice and points to empty chair] Tell him
- Martin: Why did you not defend me when I was getting called all these names?
- Therapist: You didn’t defend me.
- Martin: You sided with all the people who used to call me it.
- Therapist: You took their side. Tell him [pointing to empty chair]
- Martin: Why did you get it all wrong?
- Therapist: You got it so wrong
- Martin: Why did you say it was the way I react? Why didn’t you care about the way you made me feel?
- Therapist: I felt ….
- Martin: sad. …yeah…sad
- Therapist: I felt so sad

In EFT-AS an important component appears to be the potential for interpersonal compassion from others within the group. The benefits of this are two-fold, for one, a hurt fragile self can be soothed if another group member offers compassion and second, affective empathy can be evoked by an observing client and then used to explore their emotional response. For many autistic people, interaction with other autistic people is preferable to interacting with neurotypical people (Sinclair 2010). Further, Sinclair asserts that for him and others there is a growing realization how much autistic people have to offer each other through peer support. Most people with autistic process have early childhood experiences of being with TD peers and often it is only in adulthood that they may come to interact with other people with autistic process. It is proposed that having autistic group members offers opportunity for shared autistic attunement and as such there is the potential for an ‘autistic coach’. For example,

- Carla: Because you’re autistic. He didn’t understand you. He’s not autistic. He didn’t understand the way you think.
- Martin: No
- Therapist: And when you hear Carla saying your Dad didn’t understand you, how does that feel for you right now?
- Martin: Sad … because he didn’t know how he made me feel

Further, having EFT-AS in a small group format provides opportunities for evoking compassion responses. These can act as an addition to self-soothing. The therapist can scaffold this potential compassion reciprocity. For example,

- Therapist: Where are the emotions coming?
- Carla: I’m struggling.
- Therapist: Your emotional, I see tears.
- Carla: I’m struggling just now
- Therapist: your tears
- Carla: It’s a protective feeling I’ve got. I want to give Martin a hug [Carla looks towards Martin, who holds her gaze, they hold each others warm gaze]
- The final stage of task resolution is meaning creation and this facilitates acceptance with self-agency and marks the ending of therapy. Research findings indicate that for people with autistic process a core difficulty is in cognitive empathy, such as mental representation of others thinking (metacognition). Therefore, this cognitive empathic understanding supports the new changing narrative. For example,
- Therapist: And how do you feel about your Dad just now?
- Martin: It’s still sad that he got it wrong. I wasn’t diagnosed then and that’s why he didn’t understand me

## Conclusion

This paper has presented a developing EFT group therapy method for working with multiple layers of emotional processing and interpersonal relatedness difficulties for clients with autistic process. Through a task analytic methodology and guided by Timulak and Pascual-Leone (2014) case conceptualization framework a hypothesis has been proposed for recall of trauma-related experiences and emotion transformation. This paper set out to examine whether EFT-AS had potential value in the treatment of clients with autistic process who experience psychological distress. From this preliminary mapping a case conceptualization for working with trauma-related experience is proposed and stands as a hypothesis for future testing.
